# The fungal quorum-sensing molecule, farnesol, regulates the immune response of vaginal epithelial cells against *Candida albicans*

**DOI:** 10.1186/s12866-023-02987-7

**Published:** 2023-09-08

**Authors:** Ting Li, Zhao-Hui Liu, Lin-Yuan Fan, Zhan Zhang, Hui-Hui Bai, Feng-Juan Wang, Chen-Guang Shang, Xiao-Nan Zong, Yong Liu

**Affiliations:** 1 Department of Gynecology, Beijing Obstetrics and Gynecology Hospital, Beijing Maternal and Child Health Care Hospital, Capital Medical University, Beijing, China; 2 Department of Microecological Laboratory, Beijing Obstetrics and Gynecology Hospital, Beijing Maternal and Child Health Care Hospital, Capital Medical University, Beijing, China; 3 Department of Obstetrics, Beijing Obstetrics and Gynecology Hospital, Beijing Maternal and Child Health Care Hospital, Capital Medical University, Beijing, China; 4 Department of Gynecologic Oncology, Beijing Obstetrics and Gynecology Hospital, Beijing Maternal and Child Health Care Hospital, Capital Medical University, Beijing, China

**Keywords:** Farnesol, Vulvovaginal candidiasis, Vaginal epithelial cells, *Candida albicans*, Quorum-sensing molecules

## Abstract

**Background:**

Farnesol is a *Candida*-secreted quorum-sensing molecule of great interest as a potential antifungal agent for serious and hardly curable infections—candidiasis, especially vulvovaginal candidiasis (VVC).

**Methods:**

The effect of farnesol on cellular morphology and viability and evaluated the production of Th1 (IL-2), Th2 (IL-4), proinflammatory (IL-6), chemotactic (IL-8), and Th17 (IL-17) cytokines in the culture supernatants of vaginal epithelial cell line (VK2) were evaluated. Moreover, we tested the inhibitory effect of farnesol on *C. albicans* adhesion. Scanning electron microscopy was conducted to observe any VK2 cell ultrastructural changes.

**Results:**

Only low concentrations (≤ 50 µmol/L) of farnesol did not affect the morphology and viability of the VK2 cells (*P* > 0.05). Farnesol reduced the adhesion of *C. albicans* to the VK2 cells. When treated with farnesol, statistical elevated levels of both IL-4 and IL-17 secreted by the infected VK2 cells were present in the culture supernatants (*P* < 0.05).

**Conclusions:**

Farnesol acts as a stimulator to up-regulate the Th17-type innate immune response, as well as Th2-type humoral immunity following *C. albicans* infection. Further research is required to select the optimal therapeutic dose to develop efficacious and safe mucosal immune adjuvant for treating VVCs.

## Introduction

Vulvovaginal candidiasis (VVC) is an opportunistic fungal infection characterized by inflammation of the vulval and vaginal epithelium predominantly caused by *Candida albicans* (*C. albicans*), a commensal dimorphic fungal organism of the lower female genital tract. Indeed it has been estimated that 70–75% women will experience at least an episode of VVC during lifetime [1]. Traditionally, VVC can be treated with antifungal formulations (oral and vaginal), among which azoles are the most frequently prescribed agents, although most of them with adverse effects do not end up recurrent episodes after therapy cessation [2]. While VVC is non-lethal, its high global prevalence, increasing medical costs and profound negative impact on quality-of-life necessitate further understanding of the immunopathogenesis and treating VVC patients efficiently and prevent recurrences. The colonising yeast form of *C. albicans* is tolerated by the host’s immune system, while the invasive hyphal form triggers immune-activating signaling pathways that discriminate between commensalism vs. pathogenicity [3]. Therefore, novel therapeutic strategies are urgently required.

Opportunistic fungal infections are becoming an increasingly common cause of mortality and morbidity, now constituting a serious threat to public health worldwide, particularly for immunocompromised individuals. The innate immune system of the vaginal epithelium provides the first line of host defence against pathogenic bacteria and viruses to provide physical, chemical, and cellular barriers and protect the underlying tissues and organs [4]. Both acute VVC and Recurrent VVC (RVVC) can be attributed to specific defects in the immune system that may modulate host defence to *C. albicans*, which in turn increases the release of proinflammatory cytokines [5]. After more than two decades of research, *Candida*-specific innate host immune responses are now considered to be the most critical in combating *C. albicans* infections rather than the local or systemic adaptive immunity, including cell-mediated immunity (CMI) and humoral immunity [6]. Vaginal epithelial cells from humans and macaques appear to generate innate protection at the mucosal-*Candida* interface by inhibiting fungal growth and preventing commensal *Candida* from becoming opportunistic pathogens [6].

Quorum sensing (QS), the so-called “language of microorganisms”, designates a density-dependent signaling mechanism, wherein the microorganisms coordinate to generate a uniform activity for reproductive strategies and molecular or cellular processes and ensure the colonization of their habitats [7]. Due to the environment, microorganisms such as *C. albicans* residing within the host vagina require cell-cell communication achieved by the secretion of quorum-sensing molecules (QSMs) that sense cell density and regulate different group behaviours like morphogenesis, pathogenesis, competence, biofilm formation, bioluminescence, etc. [8]. With the increase in microbial cell concentration, extracellular QSMs accumulate in the microenvironment and once the density of signaling molecules reaches a threshold, are crucial mediators in either the expression or the repression of many functional genes. Farnesol, a vital QSM synthesized by *C. albicans* can stimulate macrophage migration, repress hyphal initiation, hypha-to-yeast transition, and inhibit biofilm formation, as well as protect *C. albicans* from oxidative stress as it induces the transcription of antioxidant-encoding genes and function as a virulence factor [9–12]. Given that fungi are eukaryotes like human host, the molecular targets that can be exploited for drug development remains limited, and their effects on human host cells still need to be further explored [13]. Recently, some studies have reported that some QSMs have been shown to exert effects on other microbial species and notably, host cells. In particular, farnesol was reported to: (i) selectively promote apoptosis and downregulate cell proliferation, angiogenesis, and cell survival of malignancies [14]; (ii) decrease the phagocytic activity of murine macrophages [15]; and (iii) act as a virulence factor of *C. albicans* by influencing innate immune cells (e.g., primary human polymorphonuclear neutrophilic granulocytes, monocytes, and monocyte-derived dendritic cells [11]).

As farnesol is a promising antifungal drug in contacting with both the host cells and *Candida*. However, the role of farnesol in the *Candida*-host interaction, particularly in vaginal epithelial cells, has not been demonstrated to date. The present study aims to explore the role of farnesol in the *Candida*-host interaction and to assess the effectiveness of farnesol for treating VVC, particularly in vaginal epithelial cells, providing potential insight into the pathogen-host signalling mechanisms that play a key role in the host immune response during VVC. Our results provide a foundation for the development of novel therapeutic strategies to treat VVC.

## Methods

### Cell lines and cell culture

The VK2/E6E7 cell line (ATCC CRL-2616) is a vaginal epithelial cell line derived from the vaginal mucosa of a healthy premenopausal females. VK2 cells were maintained in keratinocyte-serum free medium (K-SFM, GIBCO-BRL 17005-042), 100U/ml penicillin, 100U/mL streptomycin and an additional CaCl_2_ 44.1 mg/L (final concentration: 0.4 mmol/L) at 37 °C with 5% CO_2_ and 100% humidity, and were passaged every three to four days.

### Chemicals

Trans, trans-farnesol (trans, trans-3,7,11-trimethyl − 2,6,10- dodecatrien-1-ol, Sigma Chemical Co.) was dissolved in dimethyl sulfoxide (DMSO) to create a 50 mM fresh stock ), aliquoted and kept at -80 °C before use.

### Candida culture

*C. albicans* collection strains (ATCC-64,548) were grown on Sabouraud-dextrose agar in a humidified atmosphere containing 5% CO_2_ at 37 °C until the mid-exponential growth phase. For the preparation of *C. albicans* suspension, cells were resuspended in RPMI 1640 and adjusted to 1.0 × 10^6^ cells/mL. A hemocytometer (Isolab, Germany) was used for colony counting.

### Cytotoxicity assay

Cellular viability in vitro was measured by CCK-8 (Dojindo Laboratories, Tokyo, Japan) assay. VK2 cells (1.5 × 10^6^ cells/mL) were seeded into 96-well cell culture plates (Costar, Corning, N.Y.) and incubated for 24 h. Then, the VK2 cells were treated for 24 h with various concentrations of farnesol (0, 6.25, 12.5, 25, 50, 100, 200, and 400µmol/L). On the day of measurement, after carefully washing twice with phosphate-buffered saline (PBS), medium was carefully replaced on 100 µL fresh K-SFM containing 10 µL CCK-8 reagents, and incubated at 37 °C for 1 h. The absorbance at 450 nm (A_450_) was measured using a Muiltiskan GO micro-plate. Each test was repeated at least four times in quadruplexes. Results were expressed as cell viability using the following calculation:

Cell viability (%) = [A_450_ (treated)- A_450_ (blank)]/[ A_450_ (control)- A_450_(blank)] ×100.

### Determination of cytokine and chemokine levels by ELISA

The levels of Th1-immunoregulatory (IL-2), Th2- immunoregulatory (IL-4), proinflammatory (IL-6), chemotactic (IL-8), and Th17-immunoregulatory (IL-17) cytokines in the culture supernatants were determined using an Enzyme Linked Immunosorbent Assay (ELISA; eBioscience, IL-2 cat # 88-7025, IL-4 cat # 88-7046, IL-6 cat # 88-7066, IL-8 cat # 88-8086, and IL-17 cat # 88-7166) as previously described^23^. The VK2 cells (1 × 10^5^ cells/mL) were challenged with *C. albicans* (1 × 10^5^/mL) at a ratio of 1:1 for 12 h in 24-well tissue culture plates. After coculturing for 12 h, the culture medium was carefully replaced with 1 mL of different concentrations (0, 25, and 50 µM) of farnesol as described above for an additional 24 h. Then the supernatants were collected and frozen at -80 °C until use in the analysis. The concentrations of cytokines were quantified in the supernatants using ELISA in accordance with the manufacturer’s protocol. The absorbance was read at 450 nm with a Multiskan automated Microplate reader (MULTISKAN MK3, Thermo, USA). The standard curve on linear-log axis graph paper with the cytokine concentration on the x-axis (log) and absorbance on the y-axis (linear) was drawn and plotted. Cytokine levels were determined according to the standard curve. Experiments were performed in triplicate independently, and the cytokine levels were expressed as pg/mL.

### Inhibition of C. albicans adhesion on VK2 cells in vitro by farnesol

*C. albicans* cells were resuspended in RPMI 1640 at 1.0 × 10^7^ cells/mL. The VK2 cells were seeded at 1 × 10^5^ cells/mL (100:1) in 6-well tissue culture plates on a coverslip and incubated for 24 h. For pre-treatment tests, each well containing a VK2 cell monolayer was pre-incubated with 0, 50, 100 and 200 µM farnesol for 1 h at 37ºC before the *C. albicans* was added. Then, 1 mL of the *C. albicans* suspension was added and incubated for another 1 h. For treatment tests, 1 mL farnesol at different concentrations with 1 × 10^7^ *C. albicans* were applied to the VK2 cells simultaneously and then incubated for 1 h at 37 °C. For post-treatment tests, a 1 mL *C. albicans* suspension was cocultured with VK2 cells for 1 h at 37 °C. Then, 1 mL farnesol at various concentrations was added for another 1 h. Non-adherent C. albicans cells were removed after incubation. The adhesion of *C. albicans* to the VK2 cells was evaluated and counted by microscopy (magnification ×1000) following Gram staining by counting 30 consecutive VK2 cells. Experiments were performed in triplicate independently. The average number (mean) of the attached *C. albicans* per VK2 cell was assessed using the following calculation:

Adhesion inhibitory rates (%) = [Mean (control) - Mean (treated)]/Mean (control)]×100.

### Scanning electron microscope (SEM)

VK2 cells cocultured with *C. albicans* or farnesol were fixed in 2.5% glutaraldehyde for 12 h at 4 °C and stored until further use. Following a rinse three times with PBS, the samples were dehydrated using an ascending series of ethanol solutions. The samples were then air-dried overnight and loaded onto placed on stubs of aluminum and then covered with a slim layer of gold/palladium in a sputter coater. The specimens were evaluated under a Hitachi S-3400 N (LSUHSC Imaging Core) with the voltage set to 15 kV with the magnification ranging from × 50 to × 50,000.

### Statistical analysis

SPSS ver. 13.0 (SPSS, Chicago, IL, USA) was employed for data analysis. Shapiro-Wilk test and graphs (histograms and Q-Q plots) will be used to test the assumption of Gaussian distribution, and the Levene test will be used to evaluate homogeneity of variances in the different groups. The continuity data that conform to the Gaussian distribution are expressed by mean ± standard deviation, and the statistical significance for the differences between the control and test values was performed with the one-way analysis of variance (ANOVA) and LSD’s method. *P* < 0.05 indicated statistical significance.

## Results

### Farnesol may regulate the cellular morphology and viability of the VK2 cell line

We determined whether farnesol affects cellular morphology and viability. To address this question, we performed a CCK-8 assay using the VK2 vaginal epithelial cell line. We exposed the cells to a dose of 0, 6.25, 12.5, 25, 50, 100, 200, and 400 µmol/L farnesol for 24 h. CCK-8 assays indicated that higher concentrations (100, 200, and 400 µmol/L) of farnesol could inhibit the cellular viability of VK2 cells. This result was also confirmed by observations under bright inverted microscopy.

**Fig. 1:**
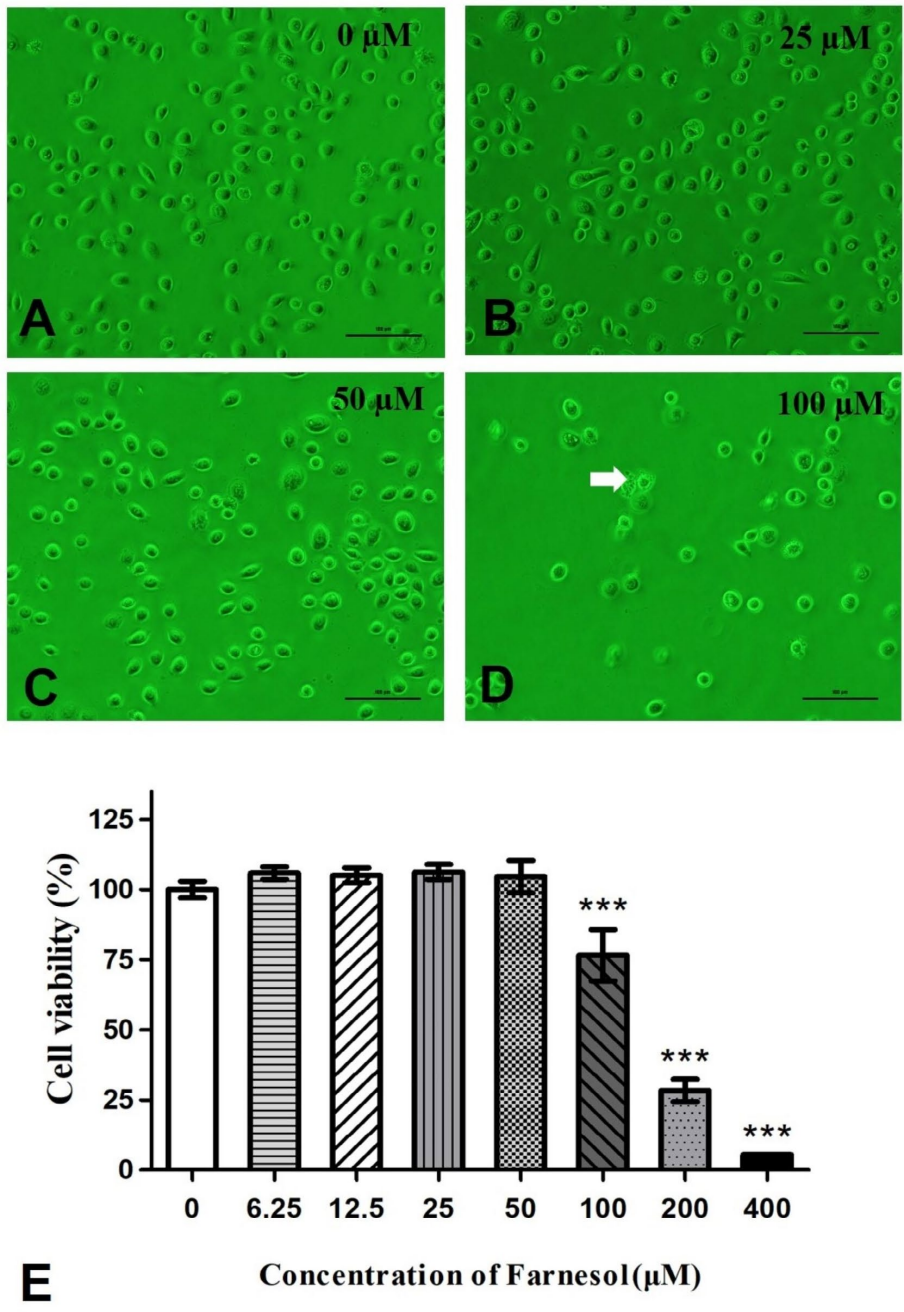
Effect of farnesol on VEC morphology. 1 **A** ~ 1**D** shows the VK2 cells treated with farnesol for 24 h through inverted phase contrast microscope (200 × magnification). The apoptotic cells treated with 100 µM is indicated by the white arrow in Fig. 1**D**, which exhibited a degraded diopter, large cytoplasms, and increased number of vacuoles. The scale bar represents 100 μm. Panel 1**E** shows the mean cell viability of the VK2 cells treated with farnesol. ***, significant difference for the epithelial cell viability (%) compared to the control (untreated) cells (*P* < 0.001)

The untreated VK2 cells exhibited a uniform shape that was either oval-shaped or elongated, with a dense round nucleus and small cytoplasm that rarely contained vacuoles (Fig. 1A **~ C**). However, when incubated with 100 µM farnesol for 24 h, the cells began to detach and degrade, developing faint nuclei, degraded diopter, large cytoplasms, and vacuoles (Fig. 1D). This inhibitory effect was confirmed by the evaluation of cell viability by CCK-8 assays (Fig. 1E).

The VK2 cells were treated with farnesol for 24 h to determine whether farnesol would adversely affect vaginal epithelial cell viability. As shown in Fig. 1E, there was a dose-dependent effect of farnesol on the vaginal epithelial. A significant (*P* < 0.001) decrease in VK2 cell viability was observed in the cultures treated with higher concentrations (100, 200, and 400 µmol/L) of farnesol, while unobvious changes was observed in the lower concentrations (0, 6.25, 12.5, and 50 µmol/L) of the farnesol-supplemented cultures (Fig. 1E).

### Farnesol may modulate cytokine release in the VK2 cell line

**Fig. 2:**
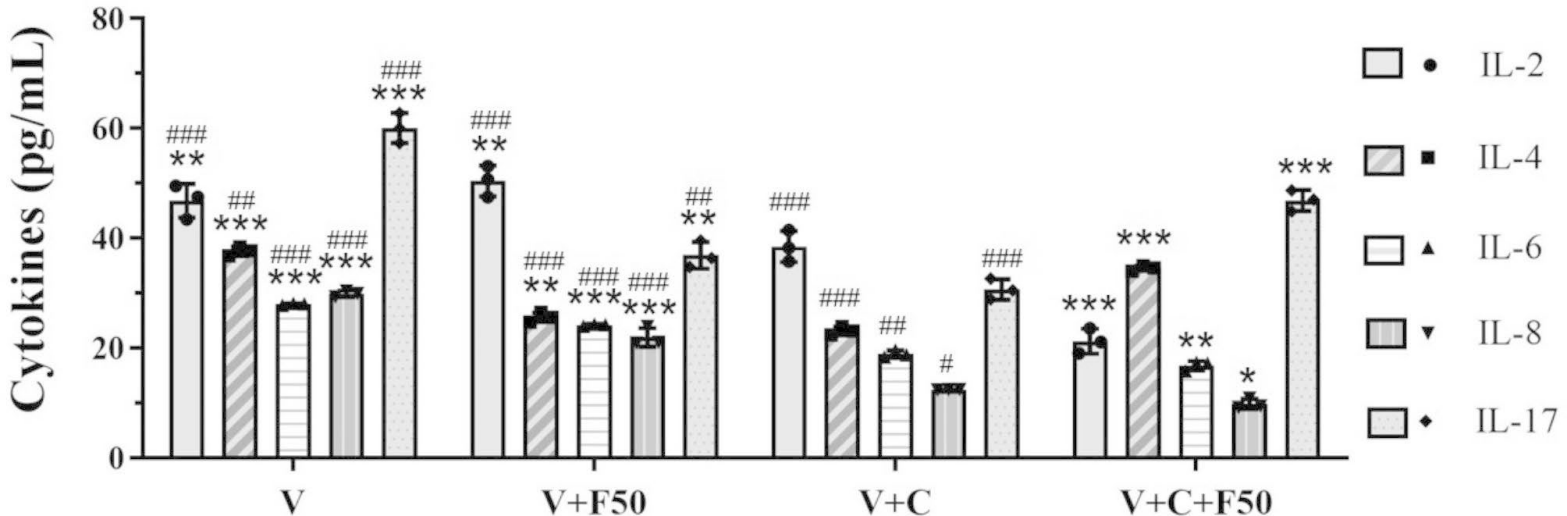
Production of Cytokines (expressed as pg/mL) by the VK2/E6E7 cells. VK2 cells cultivated alone (V), cultured with 50 µM farnesol alone (V + F50), infected with *C. albicans* for 12 h (V + C), and infected as well as treated with 25/50µM farnesol for another 24 h (V + C + F50). Cytokine levels of the supernatants were measured after 36 h for all the treatments. *, significant difference compared to the V + C group (*P* < 0.05); **, significant difference compared to the V + C group (*P* < 0.01); ***, significant difference compared to the V + C group (*P* < 0.001). ^#^, significant difference compared to the V + C + F50 group (*P* < 0.05); ^##^, significant difference compared to the V + C + F50 group (*P* < 0.01); ^###^, significant difference compared to the V + C + F50 group (*P* < 0.001)

In the present study, the baseline levels of IL-2, IL-4, IL-6, IL-8, and IL-17 production by the VK2 cells were shown in Table 1. After 24 h of co-incubation with 50 µmol/L farnesol, a known QSM produced by *C. albicans*, there was lower cytokine responses overall, with a notable down-regulation in the production of IL-4 (37.65 ± 0.85 pg/mL vs. 25.58 ± 0.88 pg/mL, *P* < 0.001), IL-6 (27.95 ± 0.15 pg/mL vs. 24.17 ± 0.20 pg/mL, *P* < 0.001), IL-8 (29.86 ± 0.55 pg/mL vs. 21.94 ± 1.70 pg/mL, *P* < 0.001), IL-17 (60.03 ± 2.71 pg/mL vs. 36.87 ± 2.45 pg/mL, *P* < 0.001) except IL-2 (46.81 ± 3.07 pg/mL vs. 50.42 ± 2.81 pg/mL, *P* = 0.148) released by the VK2 cells (Fig. 2**&** Table 1).

**Table 1:** The expression levels of cytokine of vaginal epithelial cells after co-incubation with farnesol

Group*	L-2 (pg/mL)	IL-4 (pg /L)	IL-6 (pg /L)	IL-8 (pg /L)	IL-17 (pg /L)
V	46.81 ± 3.07	37.65 ± 0.85	27.96 ± 0.15	29.86 ± 0.55	60.03 ± 2.71
V + F50	50.42 ± 2.81	25.58 ± 0.88	24.17 ± 0.20	21.94 ± 1.70	36.87 ± 2.45
V + C	38.48 ± 2.84	23.12 ± 0.76	18.91 ± 0.65	12.41 ± 0.06	30.65 ± 1.87
V + C + F25	28.86 ± 2.45	28.25 ± 0.89	17.91 ± 1.18	9.95 ± 0.43	24.81 ± 2.14
V + C + F50	21.22 ± 2.26	34.50 ± 0.49	16.80 ± 0.83	9.82 ± 0.90	46.88 ± 1.89

* V, VK2 cells cultivated alone; V + F50, cultured with 50µM farnesol alone; V + C, VK2 cells infected with *C. albicans* for 12 h; V + C + F25/50, VK2 cells infected as well as treated with 25/50µM farnesol for another 24 h. Cytokine levels of the supernatants were measured after 36 h for all the treatments

After 12 h of inoculation with *C. albicans*, all the above-mentioned cytokines released by the VK2 cells statistically decreased (0.4–0.8 fold, *P* < 0.05; Fig. 2**&** Table 1); however, when treated with 50 µM farnesol, the immune factors underwent significant changes compared with pre-medication levels, with a notable down-regulation in the production of IL-2 (38.48 ± 2.84 pg/mL vs. 21.22 ± 2.26, *P* < 0.001), IL-6 (18.91 ± 0.65 pg/mL vs. 16.80 ± 0.83 pg/mL, *P* = 0.001), IL-8 (12.41 ± 0.06 pg/mL vs. 9.82 ± 0.90 pg/mL, *P* = 0.013), while a significant up-regulation in the production of anti-inflammatory cytokines IL-4 (23.12 ± 0.76 pg/mL vs. 34.50 ± 0.49 pg/mL, *P* < 0.001) and IL-17 (30.65 ± 1.87 pg/mL vs. 46.88 ± 1.89 pg/mL, *P* < 0.001) after treatment than before (Fig. 2**&** Table 1).

### The regulation of cytokine secretion by farnesol is dose-dependent

To further explore the effect of farnesol on the cytokine secretion by vaginal epithelial cells, we used different concentrations (0, 25, 50 µmol/L) of farnesol to treat VK2 cells infected with *C. albicans* for 12 h.

**Fig. 3:**
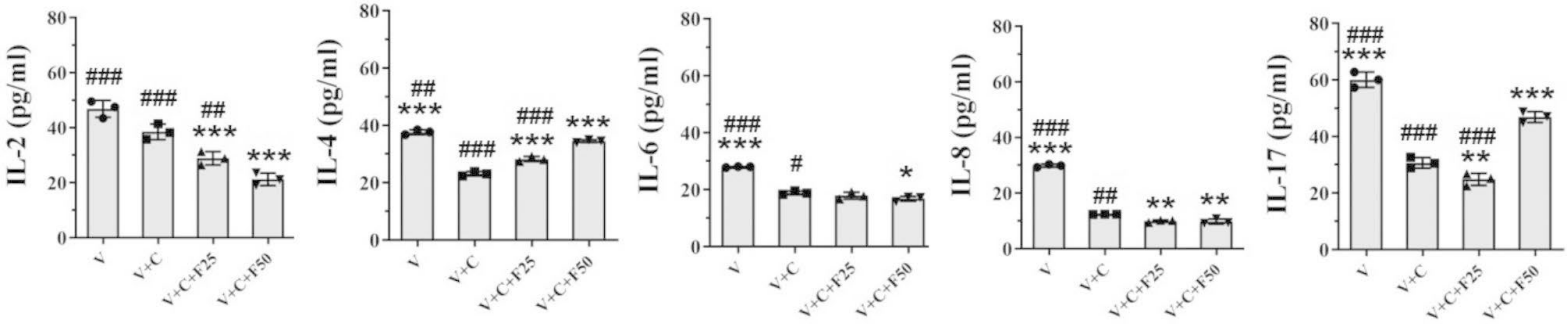
Effects of different doses of farnesol in the secretion of cytokines by the VK2/E6E7 cells. VK2 cells cultivated alone (V), infected with *C. albicans* for 12 h (V + C), and infected as well as treated with 25/50µM farnesol for another 24 h (V + C + F25/50). Cytokine levels of the supernatants were measured after 36 h for all the treatments. *, significant difference compared to the V + C group (*P* < 0.05); **, significant difference compared to the V + C group (*P* < 0.01); ***, significant difference compared to the V + C group (*P* < 0.001). ^#^, significant difference compared to the V + C + F50 group (*P* < 0.05); ^##^, significant difference compared to the V + C + F50 group (*P* < 0.01); ^###^, significant difference compared to the V + C + F50 group (*P* < 0.001). The panels shared the same data for V, V + C, and V + C + F50 as in Fig. 2

We measured the level of cytokine secretion by the infected vaginal epithelial cells from the culture supernatants and found that IL-2 gradually decreased from 38.48 ± 2.84 pg/mL (V + C) to 28.86 ± 2.45 pg/mL (V + C + F25, *P* < 0.001), and to 21.22 ± 2.26 pg/mL (V + C + F50, *P* < 0.001) following treatment with different doses of farnesol compared to the untreated infected cells. Significant differences in IL-2 level secreted by the infected vaginal epithelial cells were found between different groups, with different doses of farnesol (all *P* < 0.05, Fig. 3**&** Table 1). Our results indicated that the similar trend was also found for the production IL-6 and IL-8 (Fig. 3**&** Table 1).

In contrast, the secretion of IL-4 gradually increased from 23.12 ± 0.76 pg/mL (V + C) to 28.25 ± 0.89 pg/mL (V + C + F25, *P* < 0.001), and 34.50 ± 0.49 pg/mL (V + C + F50, *P* < 0.001) following treatment with different doses of farnesol compared to the untreated infected cells (Fig. 3**&** Table 1). The secretion of IL-17 significantly decreased from 30.65 ± 1.87 pg/mL (V + C) to 24.81 ± 2.14 pg/ml (V + C + F25, *P* = 0.011), and significantly increased to 46.88 ± 1.89 pg/ml (V + C + F50, *P* < 0.001), respectively following treatment with different doses of farnesol compared to the untreated infected cells (Fig. 3**&** Table 1).

### Farnesol inhibits the adhesion of ***C. albicans*** to the VK2 cell line

**Fig. 4:**
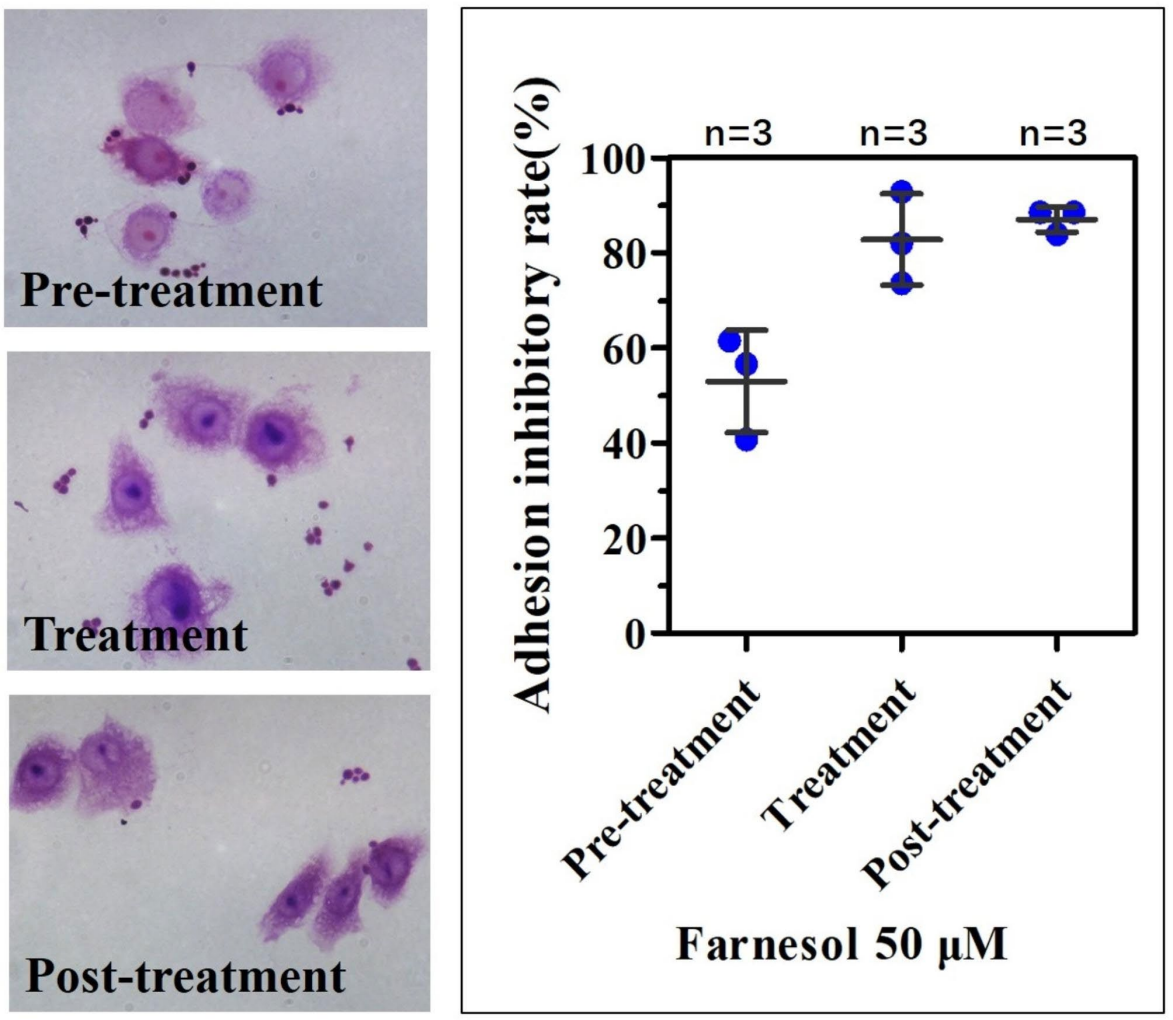
Effect of farnesol on the adhesion of *C. albicans* to human VECs. The results following the addition of 50 µM farnesol and *C. albicans* simultaneously to the VK2 cells (Treatment). The number of yeast attached to 30 consecutive cells was counted after Gram staining (100 × magnification). The control values were taken as 0% of the inhibitory adhesion rates. The data are expressed as the mean ± SD

The initial events of cutaneous candidiasis involve the adherence of blastoconidia to the surface of epithelial cells. Our results indicate that farnesol can inhibit the adhesion of *C. albicans* to VK2 cells (Fig. 4). When farnesol and *C. albicans* were added simultaneously to the VK2 cells (treated cultures), the inhibition rate of 50 mmol/L farnesol was 82.79% ± 9.63%, which was significantly higher than the controls (farnesol-untreated cultures) (*P* < 0.001; Fig. 4). When farnesol was added following a *C. albicans* challenge to the VK2 cells (post-treatment cultures), the inhibition rate of 50 µmol/L farnesol was 86.99 ± 2.65%, similar to that of the treated groups (*P* = 0.567). When farnesol was added before the *C. albicans* challenge to the VK2 cells (pre-treatment test), the inhibition rate of 50 mmol/L farnesol was 52.96% ± 10.82%, significantly higher than the controls (farnesol-untreated cultures) (*P* < 0.001), but lower than that of the treated and post-treatment groups (*P* = 0.005 and P = 0.003, respectively; Fig. 4).

### SEM revealed that farnesol restored the immunocompetence of VK2/E6E7 cells

**Fig. 5:**
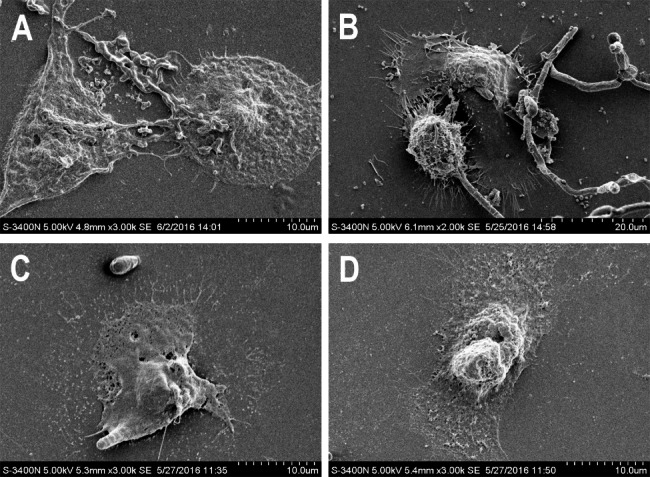
Effects of farnesol on the interaction between VECs and *C. albicans.* SEM images of normal VECs (**A**), VECs infected with *C. albicans* (**B**), and following treatment with farnesol of 50 µM for 24 h (**C** and **D**). The effect of farnesol on the yeast-to-hyphal transition of *C. albicans* (2000 ~ 3000×magnification)

To further investigate the mechanisms involved in the interaction between *C. albicans*, VK2 cells, and farnesol, we performed SEM to observe the *C. albicans* infected vaginal epithelial cells and the role of farnesol in the antifungal process and restoration of the immunologic function of vaginal epithelial cells. SEM was conducted on human vaginal epithelial cells (VK2 cells) and complete cell membrane and numerous membrane ruffling was observed on the surface of the epithelial cells (Fig. 5A). By 12 h post-inoculation, the hyphal forms of *C. albicans* were able to penetrate the VK2 cells at the apical face accompanying a cytomembrane rupture (Fig. 5B). Following farnesol (50 µmol/L) treatment for 24 h, a pseudopod-like projection from the VK2 cells was observed to actively engulf the hyphal cells (Fig. 5C) and treated cells were restored to their normal morphology (Fig. 5D). Although the *C. albicans* in farnesol-treated cell cultures exhibited decreased evidence of adhesion to the underlying cells, the production of hyphae and cellular penetration was lower compared to the cultures containing no farnesol. In contrast, after treating the cells with farnesol for 24 h, the infected vaginal epithelial cells were restored to their normal appearance and phagocytic ability as shown in Fig. 5A.

## Discussion

VVC is a type of superficial mucocutaneous candidiasis, primarily caused by *C. albicans*, and affects a significantly large number of healthy women during their childbearing years [1]. Animal models and multiple clinical studies evaluating the mechanism of VVC have shown that *Candida*-specific protection is not related to local adaptive immunity, but rather that local innate immunity may play a putative vital role in the susceptibility and resistance to infection. Vaginal epithelial cells are the main cells involved in the local innate immune response in the vaginal mucosa.

Farnesol was first identified as “acácia farnese” in the essential oils of *Vachellia farnesiana* and other aromatic plants, possessing preventive properties against various pathological implications. It is worth noting that farnesol has a great potential for applications in the food, cosmetics, and perfumery industries; moreover, farnesol show its broad pharmacological properties, such as its antifungal, antibacterial, antioxidant, anti-inflammatory, anxiolytic, analgesic, cardioprotective and neuroprotective effects [16]. However, few studies have focused on farnesol-host interactions. Therefore, in the present study, we investigated the effect of farnesol on vaginal epithelial cells. We have demonstrated that farnesol exerts a significant inhibitory effect on human vaginal epithelial cell viability and detachment ability at higher concentrations, while there was no effect on cellular morphology below a concentration of 50 µmol/L. These phenomena may be attributed to the previous findings that increased concentrations of farnesol induces cell apoptosis [17], promotes cell death [18], and inhibits hepatocyte nodule formation [19]. These results also support the findings of Saidi et al. [20] who reported that farnesol reduced normal gingival epithelial cell and fibroblast adhesion and proliferation. Moreover, this inhibitory effect appeared to be dose-dependent, as the higher the concentration of farnesol, the greater the extent of cellular damage that was observed. The findings from our study suggest that farnesol as a putative antifungal agent, should improve the effects of drugs while at the same time avoiding potential side-effects on human beings; particularly regarding classical or non-classical immune cells.

Vaginal epithelial cells produce cytokines and chemokines that play vital roles in the mucosal innate immunity of the lower female reproductive tract [21]. Active cytokine production by vaginal epithelial cells enables the rapid response to infections, thereby maintaining the sterile status of the upper genital tract. Moreover, chemokines may reinforce the subepithelial immune barrier [22]. Our results demonstrated that *C. albicans* significantly decreased the secretion of all cytokines produced by vaginal epithelial cells. This may be attributed to the ability of *C. albicans* to directly invade and kill vaginal epithelial cells, thus damaging the local innate immune function of these cells, particularly impairing the IL-17-mediated response. To simulate the effect of farnesol on vaginal innate immune mediators under in vivo conditions, VK2 cells were infected with *C. albicans* and then subsequently treated with various concentrations of farnesol for 24 h. Significantly decreased IL-4 (Th2-lineage), IL-6 (Th2-lineage), IL-8 (chemokine), IL-17 (Th17-lineage), and slightly but non-significantly increased IL-2 (Th1-lineage) were observed in uninfected cells in response to farnesol during the 24 h co-culture. Farnesol may up-regulate the induction of a protective Th1 response in vaginal epithelial cells under normal status. IL-2 is a classically representative Th1 cytokine, which is closely associated with resistance to mucosal infections by inducing Th1-type CMI. The modulation of farnesol on the uninfected vaginal epithelial cells indicates that farnesol can up-regulate local cellular immunity in the vagina under non-infectious conditions; however, it also suppresses the Th2-type humoral responses that are associated with susceptibility to infection.

Under the conditions of a *C. albicans* infection, the modulation pattern is altered as noted by the increased secretion of IL-4 and IL-17 following treatment with different doses of farnesol yet cannot reach the baseline production of aforementioned cytokines, while other cytokines were drastically decreased. Farnesol may supress the induction of a protective Th1 response and enhance the function of other protective mechanisms (i.e., the Th17 response and Th2 response in vaginal epithelial cells following infection). These findings are consistent with the research of Leonhardt et al. [11] which indicated that farnesol can activate innate immune cells (i.e., monocytes and neutrophils) but suppress cellular immune response. Based on these observations, we postulate that farnesol mediates its resistance to mucosal infections, including VVC, through Th17 cytokine responses and Th2 adaptive immune responses. In the female genital tract, Th17-driven innate immune responses have been shown to provide a protective role against *Chlamydia muridarum*, *Neisseria gonorrhoeae*, and other forms of mucosal candidiasis by inducing neutrophil migration to the site of infection [14, 23]. A defect in the immune response of IL-17-producing T cells could result in the inability to clear *C. albicans* in chronic mucocutaneous candidiasis patients [23]. However, minimal studies have explored the local and systemic Th17 cytokine responses in VVC. The present study demonstrates that farnesol elicits Vaginal epithelial cell secretion of IL-17 under conditions of an infection, which in turn can activate a Th17 response in vitro, initiating early innate immune responses against extracellular *Candida* adhesion and filamentation. Similarly, analyses of IL-4 in the supernatants from the cultures reveal that farnesol-stimulated vaginal epithelial cells secrete IL-4 under infective conditions, which can activate *Candida*-specific Th2-type humoral immunity. Nevertheless, Th2 signalling is known to dampen Th1-mediated protection, leading to susceptibility to *Candida* infection [24]. This is because relatively few *Candida*-specific antibodies (e.g., anti-*Candida*-mannoprotein [25] and anti-secreted aspartyl proteinase (SAP) antibodies [26]) can protect against vaginitis. However, other studies suggest that such antibody-mediated protection may not naturally occur in human immunity. The lack of a protective role for Th2-type humoral immunity in humans could be explained by the low abundance of these ‘protective’ mediators which do not have an effect on the fungal burden. In particular, IL-4 and *Candida*-specific antibodies may not be produced at concentrations high enough to elicit protection in the vaginal microenvironment [27]. Therefore, we postulate that farnesol acts as a stimulator or adjuvant following infection with *C. albicans*.

In the present study, we comprehensively demonstrated that farnesol was effective in reducing the adhesion of *C. albicans* to vaginal epithelial cells at low concentrations (50 µmol/L) and these results support previous research. This morphological autoregulatory molecule exerts its antifungal effect primarily through inhibition of the Ras1-cAMP pathway [28, 29] and cAMP signaling [30] via the direct inhibition of Cyr1 activity [31]. Moreover, within this concentration, farnesol exhibited no side effects regarding cellular viability of vaginal epithelial cells.

As the SEM results suggest, farnesol does not kill the yeast or disturb the physiological balance between the host and the normal microflora, but rather controls adhesion and the yeast-to-hyphal transition. Moreover, regarding aspects of the host cells, farnesol restored the normal cellular morphology and phagocytic ability, which has been considered one of the main immunological functions of vaginal epithelial cells.

limitations of the current study include the paucity of material from faithful animal models, as well as the lack of mechanism research. Further studies should establish animal model and explore the various molecular mechanisms and signaling pathways of farnesol on the epithelial response used to restore the normal vaginal structure and exert anti-fungal activity against *Candida* species.

## Conclusions

Our study is the first to evaluate the damaging effects of farnesol on vaginal epithelial cells at a high concentration, suggesting that it should be used under better-controlled concentrations (the threshold level may be 50 µmol/L in vitro) to inhibit the adhesion of *C. albicans*. Farnesol functions as a type of stimulator, similar to an adjuvant that up-regulates the Th17-axis of the innate immune responses and Th2-type humoral immunity following an infection with *C. albicans.* Further studies are required to determine the potential mechanism of farnesol for treating VVC.

## Data Availability

All the data supporting our findings is contained within the manuscript. The datasets used or analysed during the current study available from the corresponding author on reasonable request.
